# Random chromosome distribution in the first meiosis of F1 disomic substitution line 2R(2D) x rye hybrids (ABDR, 4× = 28) occurs without bipolar spindle assembly

**DOI:** 10.3897/compcytogen.v14.i4.55827

**Published:** 2020-10-09

**Authors:** Dina B. Loginova, Anastasia A. Zhuravleva, Olga G. Silkova

**Affiliations:** 1 Institute of Cytology and Genetics, SB RAS, pr. Lavrentyeva 10, Novosibirsk 630090, Russian Federation Institute of Cytology and Genetics Novosibirsk Russia

**Keywords:** immunostaining, cohesion, kinetochore microtubules, pro-spindle, monopolar orientation, phragmoplast, univalents, wheat-rye amphyhaploids

## Abstract

The assembly of the microtubule-based spindle structure in plant meiosis remains poorly understood compared with our knowledge of mitotic spindle formation. One of the approaches in our understanding of microtubule dynamics is to study spindle assembly in meiosis of amphyhaploids. Using immunostaining with phH3Ser10, CENH3 and α-tubulin-specific antibodies, we studied the chromosome distribution and spindle organisation in meiosis of F_1_ 2R(2D)xR wheat-rye hybrids (genome structure ABDR, 4× = 28), as well as in wheat and rye mitosis and meiosis. At the prometaphase of mitosis, spindle assembly was asymmetric; one half of the spindle assembled before the other, with simultaneous chromosome alignment in the spindle mid-zone. At diakinesis in wheat and rye, microtubules formed a pro-spindle which was subsequently disassembled followed by a bipolar spindle assembly. In the first meiosis of hybrids 2R(2D)xR, a bipolar spindle was not found and the kinetochore microtubules distributed the chromosomes. Univalent chromosomes are characterised by a monopolar orientation and maintenance of sister chromatid and centromere cohesion. Presence of bivalents did not affect the formation of a bipolar spindle. Since the central spindle was absent, phragmoplast originates from “interpolar” microtubules generated by kinetochores. Cell plate development occurred with a delay. However, meiocytes in meiosis II contained apparently normal bipolar spindles. Thus, we can conclude that: (1) cohesion maintenance in centromeres and between arms of sister chromatids may negatively affect bipolar spindle formation in the first meiosis; (2) 2R/2D rye/wheat chromosome substitution affects the regulation of the random chromosome distribution in the absence of a bipolar spindle.

## Introduction

Flawless chromosome segregation to daughter cells during mitotic and meiotic division is necessary to maintain the viability of organisms and their progeny. Due to the importance of these processes, cell division is controlled by multiple genes (Masoud et al. 2013, Zamariola et al. 2014, Mercier et al. 2015). Chromosome disjunction involves a multitude of processes, of importance amongst which is the formation of the division apparatus. In a plant cell, the division apparatus is comprised of membranes, kinetochores, microtubules (MTs) and actin microfilaments (Baskin and Cande 1990). MTs and actin microfilaments are elements of the cytoskeleton - a dynamic structure that changes in the course of division. MTs form bipolar spindles and interact with sister chromatids through a large protein complex - kinetochore, which is formed in centromeric regions (Perpelescu and Fukagawa 2011). As a result, MTs attach sister chromatids/homologous chromosomes to opposite poles, while chromosomes align in the spindle mid-zone and then move to the poles.

During animal cell division, the spindle is formed by special organelles - centrosomes (Walczak et al. 2010, Prosser and Pelletier 2015), in liverwort - with centrosome-like MT-organising centres, called polar organisers (PO) (Shimamura et al. 2004). Centrosomes are absent in oocytes of some animals including humans (Severson et al. 2016, Bennabi et al. 2016), as well as in cells of higher plants (Yamada and Goshima 2015).

Most of our knowledge about MTs and chromosome dynamics in higher plants was obtained while studying mitosis. MT nucleation sites are the inner surface of the plasma membrane, chromosomes and nuclear envelope (Baskin and Cande 1990, Canaday et al. 2004, Masoud et al. 2013, de Keijzer et al. 2014, Chabout and Schmit 2016, Lee and Liu 2019). The main structures formed by MTs in dividing cells during mitosis are interphase cortical or radial networks, preprophase band (PPB), prophase spindle and phragmoplast (De Mey et al. 1982, Baskin and Cande 1990, Hepler et al. 1993, Smirnova and Bajer 1992, 1994, 1998). PPB is a structure consisting of parallel MT arrays beneath the cell cortex. PPB ensures bipolarity of prophase spindles. In plants, along with PPB development, the MTs surrounding the nucleus are gradually organised into a spindle-like structure called the “prophase spindle” (“pro-spindle” or “polar caps”) (Baskin and Cande 1990, De Mey et al. 1982, Smirnova and Bajer 1992, 1994, 1998). The pro-spindle is formed with MTs that nucleate from ɣTuRCs sites and/or H1 histone complexes on the nucleus surface (Lee and Liu 2019). Motor and non-motor proteins (MAP) associated with microtubules, re-organise microtubules through sliding, cross-linking and severing into MT arrays, specific to the cell cycle phases (Chabout and Schmit 2016).

After nuclear envelope breakdown (NEB), MTs growing from polar caps become a source of interpolar spindle MTs; simultaneously, regardless of the pro-spindle during prometaphase, MT nucleation around the chromosome/kinetochore depends on the RanGTP gradient or aurora kinase. Those MTs are then organised into an overall bipolar configuration (Yamada and Goshima 2015). The mitotic spindle develops from two half-spindles, plus-ends are orientated at the mid-zone and minus-ends at the poles (Dhonukshe et al. 2006). At the mid-zone, plus-end-directed motors kinesin–5 push the poles apart by cross-linking anti-parallel microtubules and travel to their plus ends; while at the pole, minus-end-directed motors kinesin–14 draw the spindle halves together and focus the poles (Yamada and Goshima 2015). The mid-zone represents the region of overlap between the two halves of the spindle, where microtubule plus-ends terminate at chromosomal kinetochores (kinetochore microtubules) or inter-digitate in an anti-parallel manner with microtubules from the opposite pole (interpolar microtubules). The robust spindle is barrel-shaped rather than fusiform, as the pole is not tightly focused at one point; multiple kinetochore and non-kinetochore MTs are converged or cross-linked locally and, thus, multiple mini-poles are observed (Smirnova and Bajer 1992). With the start of the anaphase, sister chromatids are separated and then segregated to the pole by kinetochore MT depolymerisation, analogous to animal spindles (Yamada and Goshima 2015). The MT-based arrays assembled after sister chromatid separation are called phragmoplasts. The central factors for MT generation in the phragmoplast are ɣTuRC and augmin, whereas MAP65 is an essential MT cross-linker that ensures phragmoplast bipolarity (Yamada and Goshima 2015).

Assembly and functioning of the MT-based spindle in plant meiosis have been studied in less detail than in mitosis. In maize meiocytes, a “self-assembly” model for spindle formation was proposed (Chan and Cande 1998). Xue et al. (2019) postulate that the transition from multipolar spindles into bipolar spindles is a common process in both monocots and dicots. According to another model, a specific structure in late prophase is a ring-shaped perinuclear cytoskeleton system, which is destroyed at the beginning of the prometaphase and forms a chaotic bipolar array which is then focused and orientated into the spindle (Shamina 2005). Spindle development in the first meiosis of *Arabidopsis thaliana* (Linnaeus, 1753) is also accompanied by a specific distribution of MT arrays (Prusicki et al. 2019). A half-moon is formed in the prophase and then transforms into full-moon MT arrays surrounding the nucleus. Dense MT arrays around the nucleus, called the prophase spindle, are similar to what is observed in mitosis. When the nuclear envelope is destroyed, the prophase spindle disassembles and a robust bipolar spindle forms (Prusicki et al. 2019).

The *Arabidopsis* genome encodes two kinesin–14A genes: *Atk5/AtKIN14b* (Ambrose and Cyr 2007, Quan et al. 2008), which affects mitotic spindle pole formation and *Atk1/AtKIN14a* (Chen et al. 2002, Quan et al. 2008), which primarily affects meiotic spindle pole formation and chromosome segregation. It was found that *Dv1* is not required for the formation of bipolar spindles, but is specifically required for focusing the spindle pole to a fine point (Higgins et al. 2016). Recently, it has been observed that OsMTOPVIB, an initiation factor of homologous recombination, plays a crucial role in meiotic bipolar spindle assembly (Xue et al. 2019).

Desynaptic mutants and haploids are the target of the research of spindle assembly in the absence of bivalents. Bi-orientation of sister kinetochores in a univalent is essential for bipolar spindle formation when homologous recombination is absent in meiosis of maize asynaptic mutants and rice haploids (Chan and Cande 1998, Xue et al. 2019). Meiosis of distant hybrids is also characterised by the absence of bivalents. First-generation hybrids between species/genera of different taxa constitute a complex organism; its nucleus unites several haploid genomes. Previously, our group described four types of meiotic chromosome behaviour in hybrids (2n = 4× = 28, ABDR) between the disomic wheat-rye substitution lines 1Rv(1A), 2R(2D), 5R(5D), 6R(6A) and rye (Silkova and Loginova 2016). Two of them are significantly different from each other. The first type is a mitotic-like division in hybrids 1Rv(1A), 5R(5D) and 6R(6A) with rye. Data on MT dynamics and kinetochore architecture in univalent chromosomes indicate that the robust bipolar spindle is formed and back-to-back sister kinetochores anchor spindle microtubules followed by sister chromatid separation during the first and only meiotic division, resulting in meiotic restitution and the restoration of fertility. The second type is known as the reductional division. It is characterised by the side-by-side positioning of sister kinetochores within a random univalent distribution at anaphase I, followed by the second meiotic division. Only sterile pollen is produced as a result of such chromosome behaviour. The 2R(2D)xR genotype tends to promote reductional division in most cases (95.99±1.59 and 85.55±1.46% in different vegetations) (Silkova et al. 2007, 2011). Chromosome behaviour in the meiocytes of androgenic haploids of line 2R(2D) (Silkova et al. 2007a) was similar, 98.22 ± 1.46%, to that of the hybrids between 2R(2D) and rye (Silkova et al. 2007, 2011).

Thus, a bipolar spindle is formed in meiosis of wheat-rye hybrids when bipolar-directed kinetochores are present (Silkova and Loginova 2016), similar to asynaptic mutants and haploids (Chan and Cande 1998, Xue et al. 2019). Little is known, so far, as to how the spindle is formed and chromosomes are distributed to daughter cells in meiosis with monopolar-directed kinetochores. The study is aimed at obtaining answers to these questions. In-depth analysis was undertaken for MT cytoskeleton dynamics in meiosis of F_1_ 2D(2R)xR wheat-rye hybrids compared to MT dynamics in mitosis and meiosis of *Secale cereale* (Linnaeus, 1753) rye and *Triticum aestivum* (Linnaeus, 1753) hexaploid wheat. We did not find a robust bipolar spindle in hybrids. The presence of bivalents also does not affect the formation of a bipolar spindle. The main hybrid feature was a random distribution of chromosomes in the first division. The univalents characterised monopolar orientation and maintained cohesion. Therefore, cohesion release may affect bipolar spindle formation in meiosis. Hence, when there are no bipolar-orientated chromosomes, there is no issue with ‘release of cohesion’ and the system of motor proteins and MT kinetochores can distribute chromosomes. The minus-ends of the kinetochore microtubules were focused and formed poles. Thus, the Anaphase Promotion Complex (APC/C^CDC20^) is activated only when a bipolar spindle is formed. At anaphase I, MT bundles could connect kinetochores as bridges and kinetochores generated “interpolar” microtubules. Phragmoplast and cell plate development occurred with a delay. However, meiocytes in meiosis II contained apparently normal bipolar spindles.

## Material and methods

### Plant material

In this study, wheat cultivar *T. aestivum* cv. Saratovskaya 29 (cv S29, BBAADD, 2n=42), rye cultivar *S. cereale* cv. Onokhoiskaya (RR, 2n=14) and wheat-rye F_1_ hybrid (ABDR, 4×=28) plants were used. The parental plants of the wheat-rye hybrid included a disomic single chromosome wheat-rye substitution line (2n=42): 2R(2D) (*T. aestivum* cv. Saratovskaya 29/Novosibirskaya 67/*S. cereale* (Linnaeus, 1753) cv. Onokhoiskaya) (Silkova et al. 2006). The line was crossed as female to the diploid rye and is hereafter called 2R(2D)xR. F_1_ hybrids, wheat and rye plants were grown in greenhouse conditions at a temperature of 24/18 °C during day/night and under a day/night cycle of 16/8 h.

### Meiotic conventional analysis

For the analysis of MT dynamics in 2R(2D)xR meiosis, spikes estimated to be entering meiosis were fixed in modified Navashin’s fixative (Wada and Kusunoki 1964), which consisted of a mixture of A and B solutions (1:1). Solution A consisted of 1.1 g CdCl_2_, 10 ml glacial acetic acid and 65 ml distilled H_2_O. Solution B consisted of 40 ml CH_2_O (40%) and 35 ml distilled H_2_O. Spikes were fixed in Navashin’s fixative for two days and then the mixture was replaced with a new one. The fixed material was stored at 4 °C. Pollen mother cells (PMCs) were stained with 3% acetocarmine.

Chromosome pairing in 2R(2D)xR meiosis was examined on squashed preparations stained with 3% acetocarmine. Anthers containing PMCs at metaphase I-anaphase I were fixed in a 1:3 (v/v) mixture of acetic acid:ethanol for 24 h and stored in 70% ethanol in a refrigerator. All anthers with PMCs at metaphase I-anaphase I were analysed. Each anther was examined individually and all scorable PMCs were assayed (Table 1).

All slides were examined under a Leica DM 2000 (Leica Microsystems) microscope and images were recorded with a DFC 295 (Leica Microsystems) camera.

**Table 1.** Materials analysed in the study.

**Hybrids / rye and wheat**	**Conventional analysis**		**FISH**		**Immunostaining**		
	**Navashin’s / acetic acid:ethanol fixatives**						
	**Plants**	**Meiocytes**	**Plants**	**Meiocytes**	**Plants**	**Meiocytes**	**Mitotic cells**
2R(2D)xR	13/15	2268/7506	9	169	13	365	–
*T. aestivum* / S29	–	–	–	–	5	151	200
*S. cereale* / Onokhoiskaya	–	–	–	–	6	232	384

### Meiotic chromosome preparation and fluorescence *in situ* hybridisation

Fluorescence *in situ* hybridisation (FISH) was performed according to Silkova and Loginova (2016). Spikes were fixed in 45% acetic acid for 2 to 4 h at room temperature, anthers with meiocytes at MI-AI were selected, squashed and slides were frozen in liquid nitrogen, dehydrated through a series of alcohols with increasing concentrations of 70%, 90% and 96% and stored at –20 °C until needed. Each anther was examined individually and all scorable PMCs were assayed (Table 1).

The centromere structure of chromosomes was examined by *in situ* hybridisation using the centromere-specific probe *Aegilops tauschii* (Cosson, 1849) pAct 6–09 specific for rye, wheat, rice and barley centromere repeats (Zhang et al. 2004, Qi et al. 2013). The samples of DNA, containing the corresponding repeats, were kindly provided by Dr. A. Lukaszewcki (UCR, CA, USA). *In situ* hybridisation with labelled DNA probes was performed according to A. Houben (Houben et al. 2006). For its use as probes, the total genomic DNA of rye was labelled with biotin 16–dUTP or digoxigenin 11–dUTP by nick translation and the centromere-specific probes were labelled with digoxigenin 11–dUTP or biotin 16–dUTP by the polymerase chain reaction (PCR). The two probes were used alone or combined together in various proportions and mixed with blocking wheat DNA. Chromatin was stained using 1 mg/ml DAPI in Vectasheild anti-fade solution (Vector Laboratories). All slides were examined under an Axio Imager M1 (Karl Zeiss) microscope, images were recorded with a ProgRes MF camera (Meta Systems, Jenoptic) in the Center of Microscopic Analysis of Biological Objects (SB RAS) and processed using the Adobe Photoshop CS2 software.

### Immunolabelling

The slide preparation of mitotic and meiotic cells and immunostaining with primary and secondary antibodies was performed according to Silkova and Loginova (2016). All scorable PMCs and mitoic cells were assayed (Table 1). Root tips or anthers were fixed in fresh 8% paraformaldehyde in PBS for 2 h in a humid chamber, washed 4 × 15 min in phosphate-buffered saline (PBS) solution and digested at room temperature for 5 to 15 min in a mixture of 1% pectinase, 1% cellulase Onozuka R–10 and 1% pectolyase Y–23 dissolved in PBS. Root tips or anthers were then washed 3 × 5 min in PBS. The material was separated on poly-L-lysine-coated slides after freezing for 15 min at –70 °C and blocking for 30 min in 3% bovine serum albumin (BSA)/PBS/non-fat milk. Three primary antibodies used were anti-phH3Ser10 (1:1000; Active Motif), which specifically recognised histone H3 phosphorylated at Ser 10; anti-CENH3 (kindly provided by Dr. A. Houben, IPK Gatersleben, Germany and diluted at 1:850), which specifically recognised the centromeric histone H3 variant; and monoclonal anti-α-tubulin (Sigma, No.T5168, diluted 1:1000), which detects the α-tubulin of microtubules. The secondary antibodies to anti-phH3S10 and anti-CENH3 were anti-rabbit IgG conjugated with rhodamine (Sigma, diluted 1:100); the secondary antibody to anti-α-tubulin was anti-mouse IgG conjugated with FITC (Sigma, diluted at 1:100). Incubation with the primary antibodies was completed overnight at 4 °C. Then, slides were washed 4 × 15 min in PBS and incubated with the secondary antibody at room temperature for 1 h. After 4 × 15 min washes in PBS, the slides were counterstained with 4',6–diamidino–2–phenylindole (DAPI) and mounted in anti-fade Vectashield medium.

Slides were examined under an Axio Imager M1 (Carl Zeiss AG, Germany) microscope and the images were recorded with a ProgRes MF camera (Meta Systems, Jenoptic, Germany) with Isis software (Meta Systems, Jenoptic, Germany) or under a confocal laser scanning microscope LSM 780 NLO (Zeiss) with a monochrome digital camera AxioCam MRm (Zeiss) and ZEN software (Zeiss) in the Center of Microscopic Analysis of Biological Objects, SB RAS. The images were processed using Adobe Photoshop CS2 software.

## Results

### Dynamics of MT cytoskeleton in mitosis of bread wheat *T. aestivum* L. (2n=42) and rye *S. cereale* L. (2n=14)

**Figure 1.**
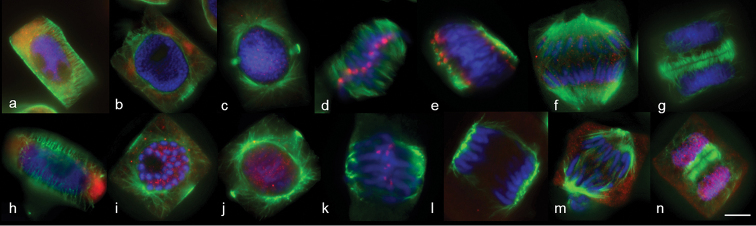
MT dynamics in wheat (**a–g**) and rye (**h–n**) mitosis. **a, h** interphase **b, c, i, j** prophase **d, k** metaphase **e, f, l, m** anaphase **g, n** telophase. Immunostaining was undertaken with anti–α–tubulin (green) and anti–histone phH3Ser10 (red) antibodies, DNA staining with DAPI (blue). Scale bar: 10 μm.

Analysis of microtubule dynamics in wheat and rye mitosis was performed using antibodies to phH3Ser10 and α-tubulin. Phosphorylation of H3Ser10 histone in mitosis has a particular dynamic (Fig. 1). At prometaphase, metaphase phH3Ser10 is localised in centromeric regions (Fig. 1d, k). It can be used to mark kinetochores to study their interaction with MTs.

MTs in wheat and rye mitosis aggregated mainly into interphase cortical or radial networks (Fig. 1a, h), pre-prophase band (Fig. 1b, i), prophase spindle (Fig. 1c, j), metaphase spindle (Fig. 1d, k) and phragmoplast (Fig. 1g, n). These structures did not differ from those described earlier for other objects (De Mey et al. 1982, Baskin and Cande 1990).

**Figure 2.**
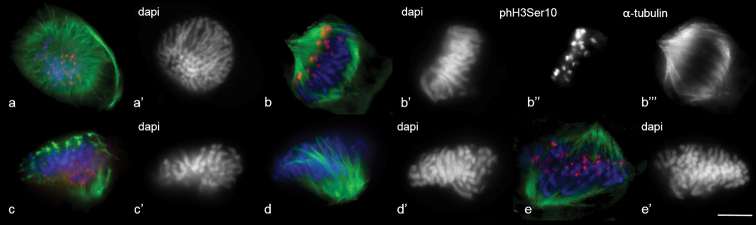
MT dynamics in wheat prometaphase. Immunostaining was undertaken with a primary antibody specific to α–tubulin (green) and histone phH3Ser10 (red). (**a**) prophase, PPB break (**b–e**) prometaphase. DAPI counterstaining (**a’–e**’). Scale bar: 10 μm.

In the early prometaphase, the pro-spindle structure changes radically after the destruction of the nuclear envelope. MT distribution changes were described in mitosis *of Haemanthus katherinae* (Martyn, 1795) (Baker) Friis et Nordal 1976 (De Mey et al. 1982). Distinct bundles of MTs are formed, a number of these bundles ending at kinetochores in the spindle mid-zone (De Mey et al. 1982). The structure of pro-spindle in wheat and rye also changed radically after the destruction of the nuclear envelope. We found cells at early wheat prometaphase where chromosomes were Rabl-orientated (Fig. 2b, c). A metaphase-like spindle was found in such cells (Fig. 2b). One pole was tightly focused at one point with this pole being located on the kinetochore side. In other cells, the regions with more intensive nucleation and tight MT arrays were also located from the kinetochore side (Fig. 2c), MTs being arranged from kinetochores towards subtelomeric regions of chromosomes (Fig. 2d). Local MT nucleation sites were also found between chromosomes with arms at the sides and subtelomeric regions, which was registered with bright massive signals of anti-α-tubulin (Fig. 2c). Inside a prometaphase spindle, chromosomes were moving and kinetochores aggregated near the spindle mid-zone (Fig. 2e).

**Figure 3.**
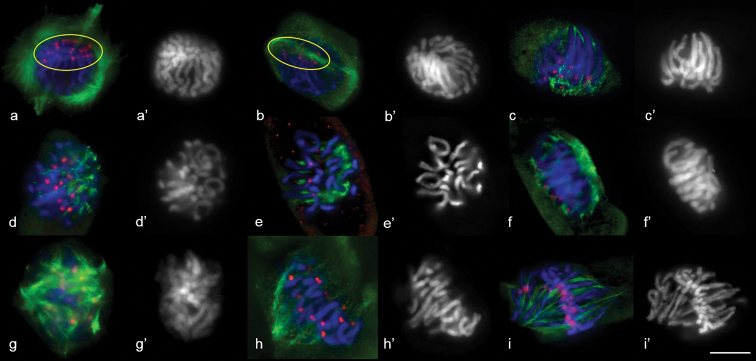
MT dynamics in rye prometaphase. **a, b** pro–spindle **c–i** MT re–organisation in prometaphase. Ovals indicate the accumulation of kinetochores. Immunostaining was undertaken with anti–α–tubulin (green) and anti–histone phH3Ser10 (red) antibodies, DNA staining with DAPI (blue). DAPI counterstaining (**a’–e**’). Scale bar: 10 μm.

Rye MT re-organisation in prometaphase differed from wheat. At the pro-spindle stage, chromosomes were Rabl-orientated (Fig. 3a, b). Rabl-orientation remained after nuclear envelope destruction (Fig. 3c). The loci of nucleation and MT growth were near kinetochores and on them, wherein MT polymerisation was unidirectional, from the bottom upwards, from kinetochores to chromosome subtelomeres (Fig. 3c). Such MT polymerisation looked like flares (Fig. 3c, d, e). As a result, the region of tighter MT arrays was registered from the subtelomere side, no tight MT arrays being found near kinetochores (Fig. 3f). Additional autonomous sites of MT nucleation emerged near kinetochores in the cells, where chromosome movement started (which was registered by the absence of Rabl-orientation). The intensity and density of anti-α-tubulin signals on such sites varied widely (Fig. 3g). MT bundles were arranged chaotically in different directions (Fig. 3g).

The later spindle had a form similar to the metaphase; however, the second pole was not developed and kinetochores were not yet bipolar-orientated (Fig. 3h). Pole convergence took place in the late prometaphase and kinetochore assembly in the spindle mid-zone was completed (Fig. 3i)

Poles in wheat and rye metaphase were transformed into several microtubule convergence centres - minipoles (Fig. 1d, k) and a mitosis-specific bipolar barrel-like (anastral) cleavage spindle was formed that aligned kinetochores on the mid-section (Fig. 1d, k). At anaphase, the kinetochore MT bundles shortened, chromosome separation began and, after chromosome separation was completed, minipoles moved closer and banded, forming tightly focused poles (Fig. 1f, m). Mitosis ended with phragmoplast formation (Fig. 1g, n) and the building of a new cell wall.

### Dynamics of MT cytoskeleton in meiosis of *Triticum aestivum* L. bread wheat (2n=42) and *Secale cereale* L. rye (2n=14)

**Figure 4.**
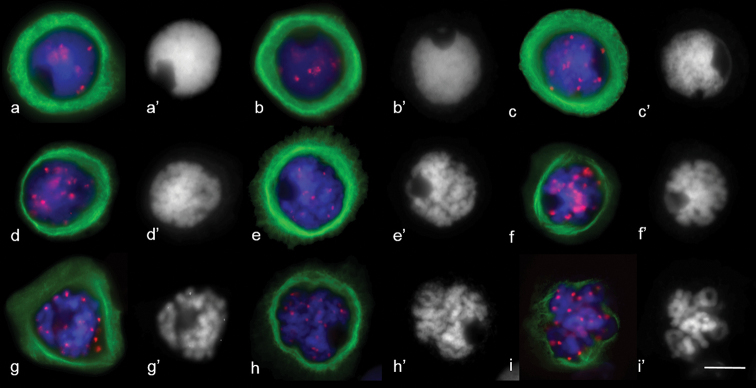
MT cytoskeleton dynamics in wheat prophase **a** Zygotene **b** pachytene **c** diplotene **d–h** diakinesis **i** prometaphase I. Immunostaining was undertaken with antibodies specific to α–tubulin (green) and CENH3 (red), DAPI counterstaining (**a’–i**’). DAPI (blue). Scale bar: 10 μm.

**Figure 5.**
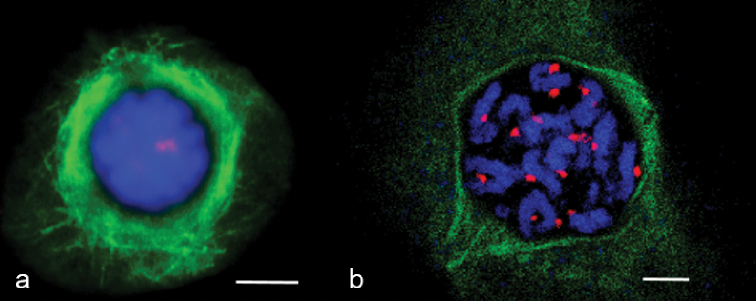
Pro–spindle formation at diakinesis in rye (**a**) and wheat (**b**). Immunostaining was undertaken with anti–α–tubulin (green) and anti–CENH3 (red) antibodies, DNA staining with DAPI (blue). Scale bar: 5 μm.

MT dynamics in wheat and rye meiosis were analysed using antibodies to phH3Ser10, CENH3 and α-tubulin. CENH3 is localised on kinetochores and phH3Ser10 on the entire chromosome in the first meiosis, while a more intensive signal is registered on the centromere at diakinesis and prometaphase. Transformation of a reticular system of MT arrays, formed around the nucleus in interphase, was observed in early prophase (leptotene, zygotene). MT polymerisation took place in different directions, a tight round-up of MT arrays formed around the nucleus (Fig. 4a) and tangential MT re-orientation resulted in development of a cortical ring near the cell membrane (Fig. 4b). The MT ring shifted to the nucleus envelope in pachytene (Fig. 4c). The MT ring remained in diplotene, while nuclei migrated to the cell edge (Fig. 4d). A perinuclear ring of microtubules formed in diakinesis (Fig. 4e), the nucleus shifting to the central position. MT structures, similar to mitotic pole caps or pro-spindles were found at diakinesis (Figs 4f, g, 5b). Rye also formed a pro-spindle-like structure at diakinesis as a result of re-organising MT arrays (Fig. 5a).

**Figure 6.**
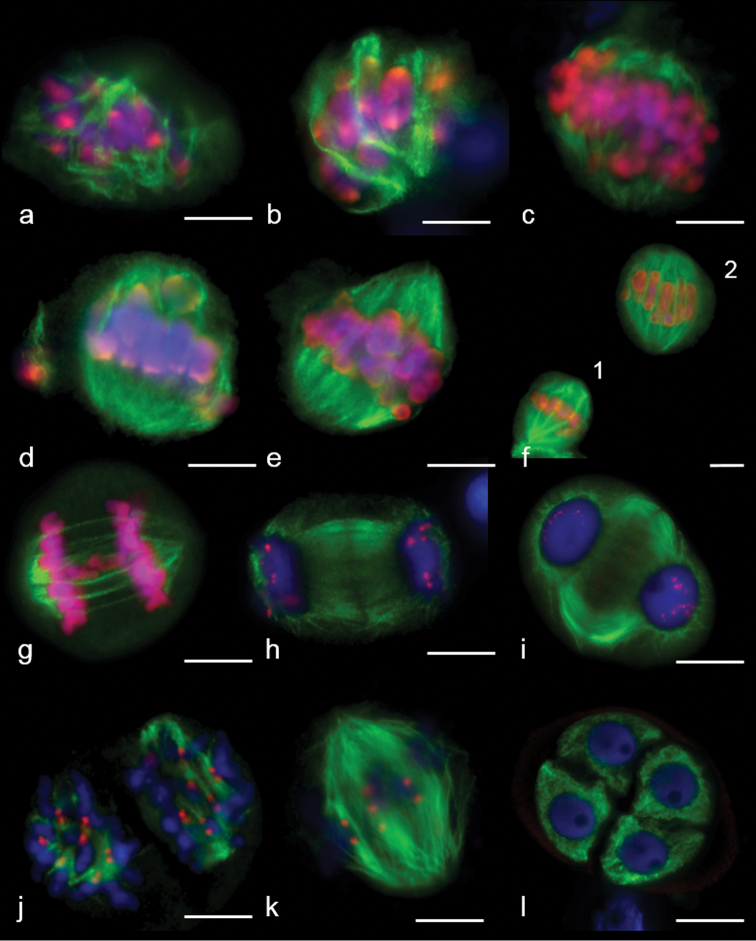
MT arrays in the first and second meiosis of wheat and rye (**j**). **a, b** early prometaphase I **c–e** prometaphase I **f** metaphase I, 1 – kinetochore MTs in the focus, 2 – interpolar MTs in the focus **g** anaphase I **h–i** telophase I **j** prometaphase II **k** one half of metaphase II **l** telophase II. Immunostaining was undertaken with antibodies specific to α–tubulin (green) and histone phH3Ser10 (red), DNA staining with DAPI (blue). Scale bar: 10 μm.

**Figure 7.**
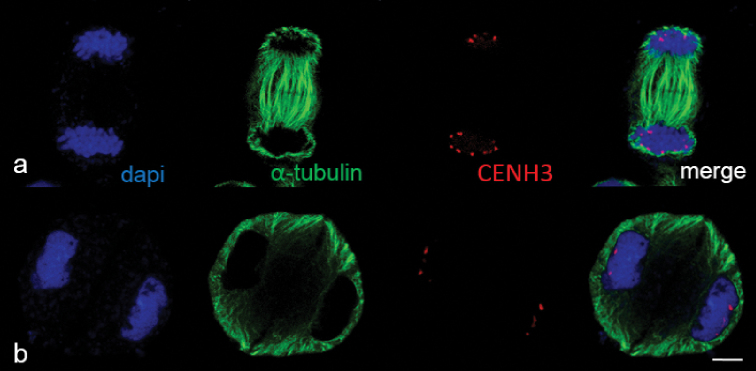
Phragmoplast expansion at anaphase I (**a**) and telophase I (**b**) in wheat. DNA was undertaken by staining with DAPI (blue). Z–stacks, confocal microscopy. Immunostaining was undertaken with anti–α–tubulin (green) and anti–CENH3 (red) antibodies. Scale bar: 5 μm.

Destruction of a nuclear envelope was accompanied by its “invagination” (Fig. 4h). The perinuclear MT ring remained, but its shape changed (Fig. 4h). After the destruction of the nuclear envelope, MTs still surrounded chromosomes along the outline of the former nucleus (Fig. 4i), while interpole and kinetochore MT bundles developed simultaneously in the prometaphase (Fig. 6a, b). Bivalents were present in meiocytes at the late prometaphase outside the metaphase plate, while homologue kinetochores formed MT bundles towards the spindle mid-zone or spindle poles (Fig. 6d, e). All bivalents at MI were positioned in the mid-region (Fig. 6f), the bipolar spindle being formed with kinetochore and interpole MTs (Fig. 6f (1, 2)). Unlike the mitotic spindle, the meiotic one had convergent poles (Fig. 6e, f). Kinetochore MT depolymerised at AI, with homologues separating to the opposite poles. Inter-regional microtubule systems could be observed in mid-late AI (Fig. 6h), which was involved in phragmoplast formation at telophase I (Figs 6h, i, 7a, b).

In prometaphase II, MTs nucleated near chromosomes and on kinetochores, where anti-phH3Ser10 was localised (Fig. 6j). Interpole and kinetochore MTs continued polymerisation in the late prometaphase and then they converged at the poles and re-organised into a bipolar spindle (Fig. 6k). Meiosis ended with cytokinesis and development of four microspores (Fig. 6l).

### Chromosome segregation in meiosis of 2R(2D)xR amphihaploids with reductional division

**Figure 8.**
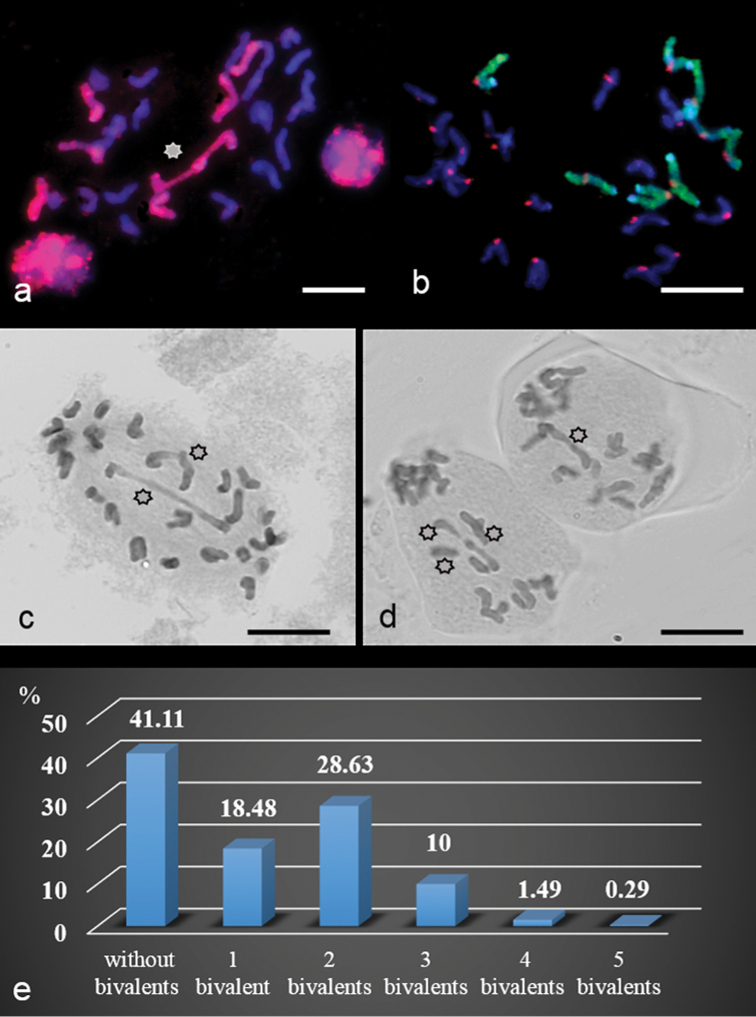
Bivalent formation in hybrids 2R(2D)xR. **a, c, d** bivalent formation (*sun*) **b** bivalent lacking **c** The distribution of meiocytes with different numbers of bivalent chromosomes. Rye chromosomes labelled red (**a**) and green (**b**), centromeres labelled red (**b**). Scale bar: 10 μm.

The main hybrid feature was a random distribution of univalent chromosomes between poles in the first division, while bivalents, whether rod or ring, lagged at the equatorial plane (Figs 8, 13c). Bivalents formed in 60.35±2.05% of meiocytes (Table 2). The average number of bivalents was 1.18±0.06 per PMC undergoing the reductional division and 0.15±0.03 per PMC undergoing equational+reductional division (Table 2). Amongst meiocytes with reductionally-dividing chromosomes, 41.11% of PMCs had no bivalents, while 18.48, 28.63, 10, 1.49, 0.29% of PMCs had 1, 2, 3, 4 and 5 bivalents, respectively (Fig. 8).

**Table 2.** Bivalent formation according to chromosome division type in 2R(2D)xR hybrids.

The mean number of bivalent per cell			The percent of meiocytes with bivalent chromosomes		
reductional	equational + reductional	overall	reductional	equational + reductional	overall
1.18±0.06	0.15±0.03	1.09±0.05	60.35±2.05	3.06±0.9	63.41±1.69

**Figure 9.**
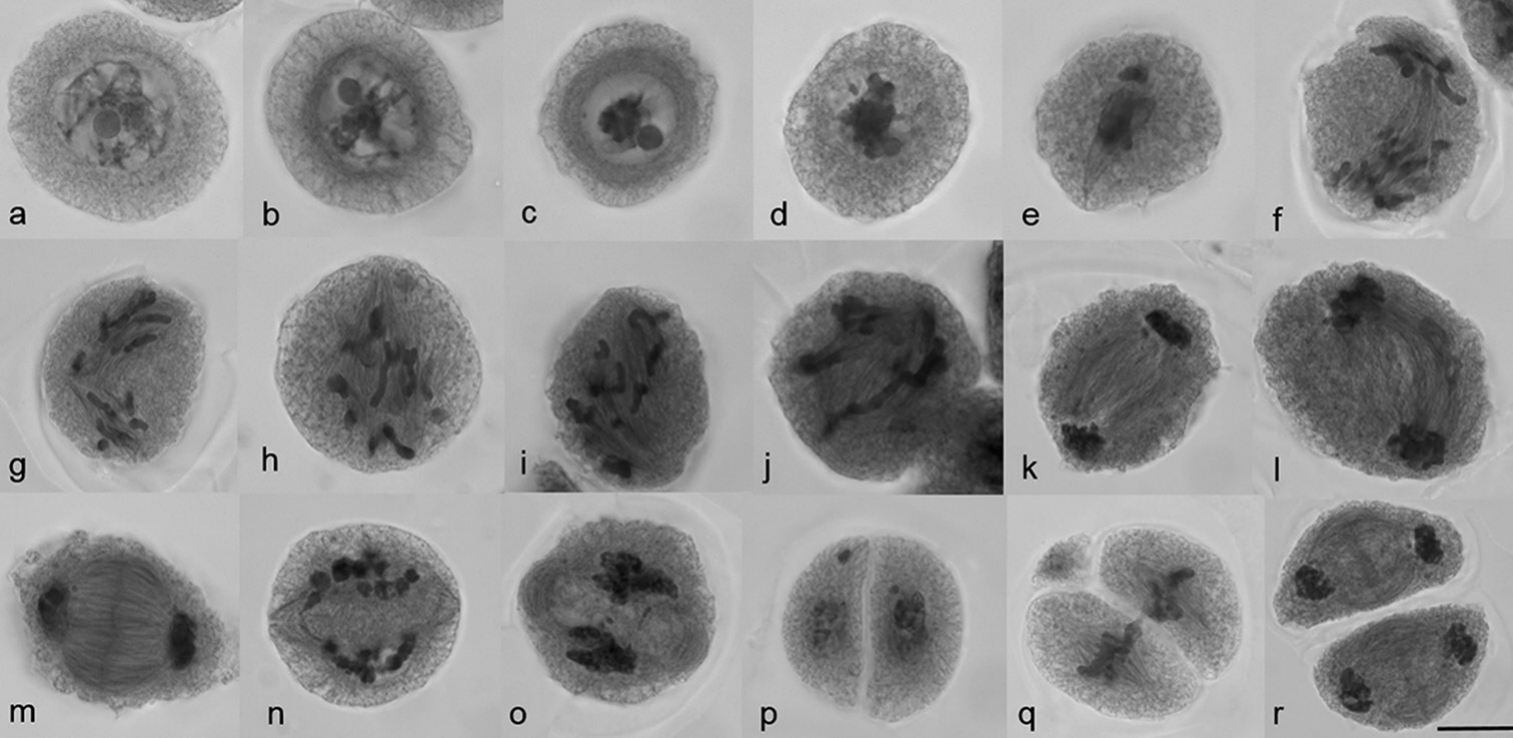
MTs dynamics in meiosis of hybrids 2R(2D)xR. **a–c** prophase I **d** prometaphase I **e** metaphase I **f–j** anaphase I **k–o** telophase I **p** interkinesis **q** metaphase II **r** telophase II. Scale bar: 10 μm.

To understand the meiotic mechanisms of chromosome divergence in hybrids, the formation and functioning of the division apparatus are analysed. An analysis of MTs dynamics in meiosis, using Navashin fixation, showed MT re-organisation in pachytene: first, MTs were positioned cortically (Fig. 9a) and then perinuclear (Fig. 9b); a tight MT perinuclear ring was formed in diplotene (Fig. 9c). The ring could not be visualised after the destruction of the nuclear envelope; isolated MT arrays were observed (Fig. 9d). At metaphase I, chromosomes were in the cell centre in close contact with each other, but without the formation of the classic metaphase plate (Fig. 9e). MT developed bundles, but no bipolar spindle was found (Fig. 9e). At anaphase I, chromosomes were mostly arranged into two groups (Fig. 9f, g). The spindle had a curved form in 66.65±3.35% meiocytes (Table 3) (Fig. 9f, g, l) and the spindle was straight (as usual) in 29.35±3.0% (Table 3) (Fig. 9h, k) or chaotic MT bundles were present in a cell (Fig. 9i, j). Phragmoplast developed at the end of the first division (Fig. 9m-o), cytokinesis occurred and a cell wall was formed (Fig. 9p). In some cases, a cell plate was not observed, chromosomes remained condensed and telophase groups were not formed (Fig. 9n). A MT bipolar spindle was assembled in the second division (Fig. 9q), phragmoplast developed after chromosome separation (Fig. 9r) and cytokinesis occurred.

**Table 3.** The percentage of meiocytes with different forms of spindle in the first meiosis of 2R(2D)xR hybrids.

Curved spindle	Straight spindle	3–poles spindle
66.65±3.35	29.35±3.0	0.68±0.41

Immunostaining with anti-α-tubulin revealed the specifics of MT dynamics in the first meiosis. Kinetochores were visualised using antibodies to CENH3 as a means to distinguish chromosomes from one another. Given that phosphorylation of histone H3Ser10 residue in plants is cell-cycle dependent and related to cohesion maintenance, we used anti-H3Ser10ph as a marker of cohesion upon sister chromatid segregation and to visualise meiotic stages.

**Figure 10.**
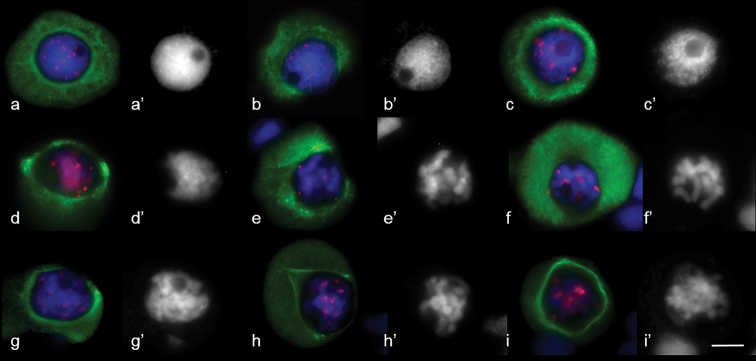
MT cytoskeleton dynamics in 2R(2D)xR hybrids prophase. **a, b** zygotene **c** pachytene **d** diplotene **e–h** diakinesis **i** prometaphase I. Immunostaining was undertaken with anti–α–tubulin (green) and anti–CENH3 (red) antibodie,. DAPI counterstaining **(a’ – i’).**DAPI (blue). Scale bar: 10 μm.

At the early stages of prophase (leptotene-zygotene), meiocytes contained networks of cytoplasmic MTs (Fig. 10a). These MTs appear to be randomly arranged and tangential to the nuclear surface, a tight narrow perinuclear ring forming near the nuclear envelope (Fig. 10a). Both tight and more disperse cortical MT arrays were found at pachytene in different meiocytes (Fig. 10b, c).

The presence of a bright α-tubulin halo around the nucleus was a common feature of meiocytes at diplotene (Fig. 10d). MTs were arranged unevenly inside the halo, while autonomous circular bright and tight α-tubilin signals were found. As diakinesis progressed, MT arrays demonstrated different shape, density and distribution (Fig. 10e – h). MT arrays crossed over each other or were organised in a parallel manner (Fig. 10e). Before the nuclear envelope breakdown, some meiocytes contained noticeable triangular MT arrays with one focused pole (Fig. 10h). Destruction of the nuclear envelope at diakinesis was accompanied by its invagination (Fig. 10i). The perinuclear MTs ring changed its shape. The specific feature of prophase substages in hybrids was the migration of nuclei to the cell edge.

**Figure 11.**
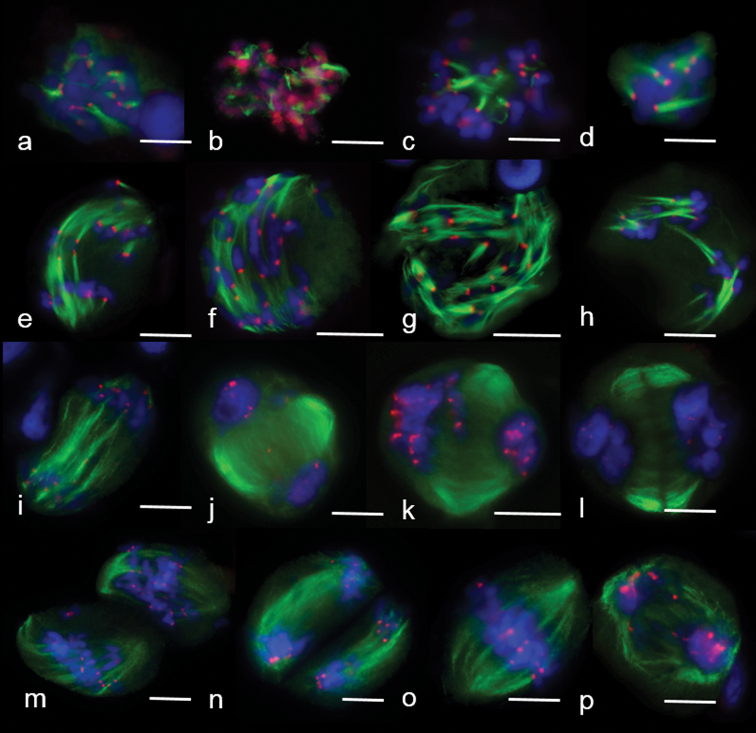
MT arrays in the first and second meiosis of 2R(2D)xR hybrids. **a, b** early prometaphase I **c, d** metaphase I **e–h** anaphase I **i** the late anaphase I **j–l** telophase I **m** metaphase II **n** anaphase II **o** one–half of meiocytes at metaphase II **p** one–half of meiocytes at anaphase II. Immunostaining was undertaken with antibodies specific to α–tubulin (green) and CENH3 (red) **(a, c–t)** and **(b)** histone phH3Ser10 (red), DNA staining with DAPI (blue). Scale bar: 10 μm.

At prometaphase, the microtubules appeared to nucleate from multiple sites in the cells and surround the chromatin (Fig. 11a, b). Microtubules even appeared to emanate directly from the chromosome surface. Later, we detected large branched bundles of microtubules associated with kinetochores (Fig. 11c, d). Chromosomes are not aligned at the metaphase plate; they were located close to each other in the centre of the meiocyte (Fig. 11c, d). MT bundles elongated and attempted to cross-link (Fig. 11c, d).

**Figure 12.**
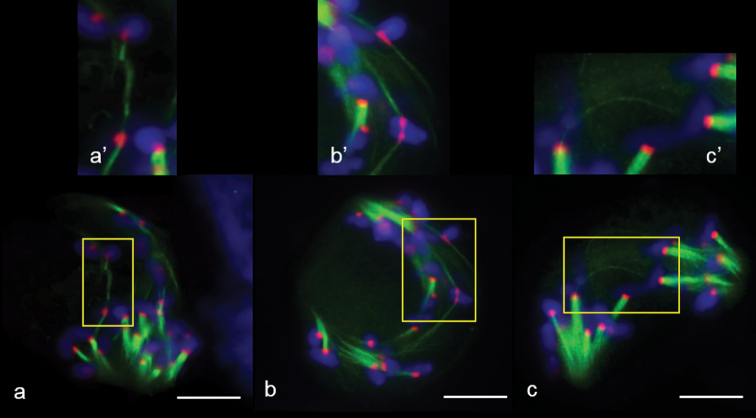
Bipolar and monopolar kinetochore orientation at anaphase I in meiosis of 2R(2D)xR hybrids. **a** kinetochores linked by microtubules **b** bi–polar kinetochore orientation **c** thin kinetochore MT bundle – like interpolar MT bundle. Immunostaining was undertaken with anti–α–tubulin (green) and anti– CENH3 (red) antibodies, DNA staining with DAPI (blue). Scale bar: 10 μm.

**Figure 13.**
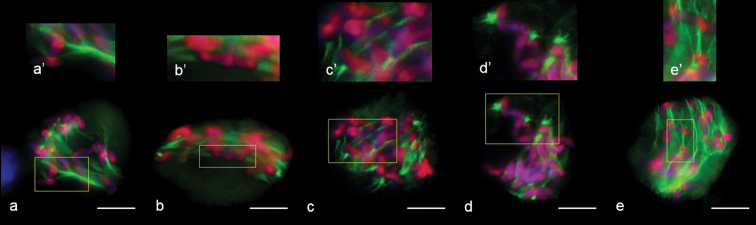
Patterns of MT arrays distribution in the first meiosis of 2R(2D)xR hybrids. **a** cross–link of three MT kinetochore bundles, one of them with bipolar orientation **b** bivalent lies in the metaphase plate **c, e** MT bridges between kinetochores **d** kinetochores as sites of MT nucleation. Immunostaining was undertaken with antibodies specific to α–tubulin (green) and histone phH3Ser10 (red). Scale bar: 10 μm.

We identified meiocytes where chromosomes were divided up into groups as anaphase I (Fig. 11e–h). Since interpolar MTs were not found (Fig. 12c), the standard bipolar spindle was not formed and we marked the sites where kinetochore MTs focused as “pole”. At this stage, MTs’ minus-ends attempted to focus or were “searching” the pole site (Fig. 11e, f, h). Although MT bundles could connect kinetochores as bridges, perhaps they compensated for the absence of inter-pole MTs (Figs 11a, 12c, e). MT kinetochore bundles cross-linked and grew to the “pole” (Fig. 12a). In some cases, MT bundles did not cross-link and were not focused and chromosomes were not grouped (Fig. 11g). Due to the presence of bright strong signals of α-tubulin, kinetochores seem to be MT nucleation sites (Figs 12a–c, 13a–d). In all described cases, chromosome kinetochores were monopolar-orientated and, at anaphase I, we observed single tight pin-pointed anti-CENH3 signals, matching the integrated kinetochore of sister chromatids (Figs 11e–h, 12a–c). We did not see changes in monopolar orientation of kinetochores to bipolar (re-orientation). Sometimes, chromosomes with two anti-CENH3 signals, from which MT bundles grew to opposite poles, were found in meiocytes (Figs 11e, 12b). Such chromosomes were delayed on the mid plane after the divergence of univalents with a monopolar orientation. If bivalents were present in meiocytes, first, univalents were arranged between “poles” while the bivalent was delayed in the spindle mid-zone (Fig. 13b).

Other specifics of reductional chromosome separation include the absence of division of sister chromatids at anaphase I, which was identified by the absence of “x” shaped chromosomes. phH3Ser10 localisation during chromosome separation in meiosis I was characterised by more intensive staining of centromeres compared to chromosome arms (Fig. 13). After chromosome separation, between telophase groups, inter-zonal MTs assembled (Fig. 11i). It seems they were formed by kinetochore microtubules (Fig. 11i). Phragmoplasts without a formed cell plate were found (Fig. 11j); however, cytokinesis occurs in most meiocytes (Fig. 11k, l). Meiocytes were capable of progressing through meiosis II. These cells also contained apparently normal bipolar spindles (Fig. 11m–p); however, chromosomes did not always segregate properly.

## Discussion

### Specifics of the MT-based spindle assembly in mitosis of rye and wheat

In mitosis after the nuclear envelope breakdown (NEB), MTs growing from polar caps become a source of MTs of the interpolar spindle. At the same time, regardless of the pro-spindle, MTs nucleate near the chromosomes/kinetochores during the prometaphase (nucleation depends on RanGTP gradient or aurora kinase) and those MTs are then organised into an overall bipolar configuration (Yamada and Goshima 2015). We discovered asymmetric MT arrangements after NEB in the prometaphase of rye and wheat mitosis. The common feature at the onset of prometaphase was nucleation and MT polymerisation near the kinetochores. At this stage, chromosomes maintained their Rabl-orientation. Wheat formed a pole from the kinetochores side and MT polymerisation was towards chromosome telomeres. It seems that chromosome relocation and continued spindle assembly took place simultaneously. Kinetochores were also the site of MT nucleation in rye; further MT polymerisation had a flame-like shape. As a result, a tight MT array formed on the telomere side. Subsequently, the spindle assembly occurred similarly to the wheat assembly. Such asymmetry in bipolar spindle assembly was registered using live imaging of microtubules in *A. thaliana* (Komis et al. 2017). Pro-spindle re-organisation began before PPB disruption, MT re-arrangement started from one PPB side (one half of spindle) and then the second half of the spindle assembled simultaneously with PPB disassembly (Komis et al. 2017, video 8). The same time was needed to align chromosomes in the spindle mid-zone and form a robust bipolar spindle as for assembling a prometaphase bipolar spindle. Multipolar, apolar and monopolar prophase spindles are relatively common in *Haemanthus katherinae* (Smirnova and Bajer 1992). During the prometaphase, these three types of spindle differentiate invariably into the bipolar metaphase spindle (Smirnova and Bajer 1992).

### Meiotic spindle assembly in rye and wheat

Meiotic spindle assembly was studied for several species of dicotyledon and monocotyledon plants (Chan and Cande 1998, Shamina 2005, Xue et al. 2019). In maize meiocytes, a ‘self-assembly’ model for spindle formation was proposed (Chan and Cande 1998). According to the model, MTs initially appear around the chromosomes during the prometaphase, followed by self-organisation of the MTs into a bipolar spindle (Chan and Cande 1998). In both monocots (rice, maize) and dicots (tobacco, *Arabidopsis* Heynhold, 1842), a multipolar spindle has been found at early metaphase I and this re-organises into a bipolar spindle at the metaphase I (Xue et al. 2019). The authors postulate that the transition from multipolar spindles into bipolar spindles is a common process in both monocots and dicots (Xue et al. 2019). According to another model, the MTs’ perinuclear ring is the structure specific to the late prophase (Shamina 2005). At the beginning of the prophase, the ring degrades. MTs interact with chromosome kinetochores and with each other and form a chaotic bipolar array which is then focused and orientated into the spindle (Shamina 2005). Spindle development in the first *A. thaliana* meiosis is accompanied by a specific arrangement of MT arrays (Prusicki et al. 2019). Half-moon MT arrays are found in late prophase and these are later transformed into full-moon ones surrounding the nucleus. The tightening of MT arrays around the nucleus was called the “prophase spindle”, similar to what is observed in mitosis. When a nuclear envelope degrades, the prophase spindle is then disassembled and a robust bipolar spindle is formed (Prusicki et al. 2019).

We found in wheat that the prophase MTs arranged similarly to that which was described by Shamina (2005). A perinuclear ring also formed at diakinesis; then, however, the MTs that form the ring re-orientated and structures that looked like mitotic pole caps or pro-spindle developed. In rye diakinesis, MTs arrays formed a diamond-shape prophase spindle, similar to the pro-spindle of *A. thaliana* (Prusicki et al. 2019). Rye and wheat showed simultaneous development of interpolar and kinetochore MT bundles in the prometaphase. We did not find multipolar spindles either in the later prometaphase or in the early metaphase. Perhaps, assembly of the meiotic spindle in wheat and rye occurs similarly to the mitotic spindle assembly, while the pro-spindle “poles” mark the sites of future poles of the metaphase spindle. In plant mitosis, ɣ-tubulin exhibits bipolar localisation from prophase to anaphase (Binarová et al. 2000). TPX2 participates in the pro-spindle formation; it concentrates in the polar caps (Vos et al. 2008).

### The function of Anaphase Promotion Complex APC/C^cdc20^ fails in 2R(2D)xR hybrids

Chromosome separation in normal meiosis I has its specifics. Single DNA replication occurs in the S-phase, when DNA copies (sister chromatids) are captured by a ring-shaped protein complex called cohesin. In the meiosis I prophase, homologues pair and become joined by a synaptonemal complex and then exchange DNA reciprocally during crossover recombination, forming chiasms (Petronczki et al. 2003, Zamariola et al. 2014, Ohkura 2015, Zickler and Kleckner 2016). MTs of a meiotic spindle establish attachments to the bivalents. Two sister kinetochores of each homologue are captured by the same pole, not by opposite poles as occurs during meiosis II and mitosis. As soon as bipolar homologue attachment takes place and bivalents align on the metaphase plane, meiotic cohesion between chromosome arms is destroyed, allowing the homologues to segregate at the opposite poles (Petronczki et al. 2003, Ohkura 2015). Two sister kinetochores of each homologue remain tethered by the surviving cohesin complexes before meiosis II. In meiosis II, sister kinetochores are captured by the opposite poles and move apart when the remaining cohesin complexes are destroyed.

Random distribution of chromosomes in meiosis I of 2R(2D)xR hybrids was characterised by monopolar kinetochore orientation and their side-by-side geometry, as well as maintained cohesion between sister chromatids in the anaphase. Normally, absence of bipolar attachments of kinetochores and their tension between the poles cause an anaphase delay due to insertion of a spindle assembly checkpoint (SAC). Components of this complex include evolutionally conservative proteins Chromosome Passenger Complex (CPC): the Ser/Thr kinases monopolar spindle 1 (MPS1), Aurora B and Budding Uninhibited by Benomyl 1 (BUB1) and BUB3 and the non-kinase components Mitotic Arrest Deficient 1 (MAD1), MAD2, BUB1 Related kinase 1 (BUBR1), Cell Division Cycle 20 (CDC20) (Kang and Yu 2009, Lara-Gonzalez et al. 2012, Zamariola et al. 2014). All these proteins localise to unattached kinetochores and generate a kinetochore signal that inhibits the anaphase-promoting complex/cyclosome. APC/C^CDC20^ remains inactive until all sister chromatids are attached to the mitotic spindle and under tension indicating an equal alignment of the chromosomes in the metaphase plate (Kang and Yu 2009, Lara-Gonzalez et al. 2012, Wijnker and Schnittger 2013). APC/C is activated twice in meiosis: at anaphase I and anaphase II (Yamamoto et al. 2008).

Absence of APC/C activity can be one of the reasons for chromosome separation with monopolar orientation and maintaining cohesion with sister chromatids. APC/C can be inactive due to the absence or disrupted signal transfer from SAC proteins. Homologues of SAC proteins are involved in plant meiosis, including maize MAD2 (Yu et al. 1999), rice BRK1 (BUBR1) (Wang et al. 2012) and *A. thaliana* Aurora kinases (Demidov et al. 2014). MAD2 localises on an outer kinetochore and is necessary for sensing the amount of tension at a kinetochore (Yu et al. 1999). MAD2 plays the key role in SAC, since it bonds with CDC20 on a kinetochore with abnormal attachment to MTs (Kang and Yu 2009, Lara-Gonzalez et al. 2012). It is known that to activate APC/C, one of the two CDC20 or Cdh1 co-factors is necessary (Lara-Gonzalez et al. 2012). In wheat, the MAD2 gene is mapped on the chromosomes of the 2^nd^ homoeologous group (Kimbara et al. 2004). It is assumed that MAD2 protein is involved in SAC control in wheat mitosis as, in the course of colchicine treatment of dividing cells, MAD2 remains on kinetochores in the metaphase, but normally it is absent (Kimbara et al. 2004). Perhaps, the genotype of the disomic substitution line 2R(2D), used in rye crossing, has the MAD2 gene. In this case, MAD2 protein in the complex with CDC20 is presumably localised on kinetochores and, in the absence of kinetochore tension due to its monopolar orientation, the MAD2/CDC20 complex is maintained and APC/C is not activated. As a result, the cohesion between the kinetochores and arms of sister chromatids does not cleave.

Otherwise, release of cohesion may also be impossible due to activation of Aurora B kinase. Aurora B controls multiple aspects of cell division and plays a key role in bipolar spindle assembly (Zhang and Dawe 2011, Joukov 2011). H3Ser10 histone in plants is a substrate of AtAurora3 (Kawabe et al. 2005) and it is shown that phH3Ser10 and AtAurora3 are localised in centromeric regions of mitotic chromosomes (Demidov et al. 2005). For most plant species, phosphorylation of H3Ser10 is typical for only pericentromeric regions in mitosis and the second division of meiosis and along the entire length of the chromosomes in the first meiotic division (Loginova and Silkova 2017). Based on these data, a conclusion is reached that localisation of phH3Ser10 in plants is associated with maintained cohesion (Kaszas and Cande 2000, Manzanero et al. 2000, Houben et al. 2007). Therefore, phosphorylation of H3Ser10 can indicate involvement of Aurora3 (Aurora B) in maintaining cohesion on kinetochores. In 2R(2D)xR hybrids, maintaining cohesion was identified indirectly by localisation of phH3Ser10 along all chromosomes (Fig. 13). phH3Ser10 localisation during chromosome separation in meiosis I was characterised by more intensive staining of centromeres compared to chromosome arms (Fig. 13). Probably, Aurora-kinase is localised on monopolar-orientated kinetochores and cohesion is maintained. It turned out that the CDC20, APC/C co-factor, regulates Aurora localisation on chromosomes in meiosis of Arabidopsis (Niu et al. 2015). Abnormal distribution of H3Ser10 and H3Thr3 histones, phosphorylated with Aurora-kinase, is found in meiocytes of *cdc20.1* mutant (Niu et al. 2015). In meiosis of *cdc20.1* mutants, chromosomes align asynchronously and segregate unequally and the metaphase I spindle has aberrant morphology (Niu et al. 2015). These findings indicate the involvement of CDC20.1 in SAC-dependent segregation of meiotic chromosomes (Niu et al. 2015).

On the other hand, why are monopolar-orientated kinetochores in 2R(2D)xR hybrids unable to re-orientate bipolarly at metaphase I? Bi-orientation of sister kinetochores in a univalent is essential for bipolar spindle formation when homologous recombination is absent (Chan and Cande 1998, Xue et al. 2019). We found bipolar orientation in hybrids with 1Rv(1A)xR, 5R(5D)xR and 6R(6A)xR lines (Silkova and Loginova 2016). In 2R(2D)xR meiocytes, there were only rare univalents on the mid section with two CENH3 signals and bipolar orientated MT bundles or univalents near poles had extended kinetochores (dumb-bells) and two attached MT bundles. What can affect the inability of kinetochores for bipolar re-orientation? The reasons for the monopolar orientation in 2R(2D)xR hybrids may be the formation of the MIS12 – NDC80 bridge at the kinetochore (Li and Dawe 2009) plus the protection of centromeric cohesion of univalents by SGO1 from destruction (Kitajima et al. 2004, Zamariola et al. 2013). SGO1 recruits PP2A at centromeres to dephosphorylate REC8, making it resistant to separase cleavage.

Apart from CPC proteins, γ-tubulin complex protein 3–interacting proteins (GIPs) are essential for the proper recruitment and/or stabilisation of centromeric proteins, as well as for centromeric cohesion in somatic cells (Batzenschlager et al. 2015).

The bipolar spindle in meiosis of asynaptic mutants of maize and rice haploids is formed regardless of the presence of bivalents (Chan and Cande 1998, Xue et al. 2019). On the contrary, in 2R(2D)xR hybrids, a bipolar spindle is not formed in the presence of bivalents. Therefore, cohesion release, but not the presence of bivalents, may affect bipolar spindle formation in meiosis. Cohesion between univalent arms and sister chromatids of bivalents can be maintained due to the CDC20 co-factor related to CPC proteins, while cohesion in the centromeric region may be protected by SGO1.

### Chromosome segregation without bipolar spindle in 2R(2D)xR hybrids

MT arrangements throughout prophase in 2R(2D)xR hybrids deviated from the norm. The main features were nucleus migration at all stages of prophase and uneven distribution of cortical MTs. MTs formed a triangular pro-spindle in diakinesis and bright tight signals of α-tubulin were localised in the triangle angles, perhaps at the sites of MT nucleation. At the prometaphase, the microtubules appeared to nucleate from kinetochores and to surround the chromatin. MT bundles were evident from the chromosome mass at metaphase I. Zhang and Dawe (2011) have also observed small kinetochore fibres in barley (*Hordeum vulgare* Linnaeus, 1753), formed immediately after NEB, suggesting that plant kinetochores may initiate their own kinetochore fibres early in the prometaphase. Many proteins involved in MT nucleation and spindle assembly interact with kinetochores. It has been demonstrated that RanGAP1 associates with kinetochores in mitosis (Lipka et al. 2015). TPX2 may both catalyse new MT nucleation from ɣ-TuRCs around chromatin in a GTP-Ran dependent pathway and stabilise kinetochore fibres (Aguirre-Portoles et al. 2012). In meristem cells of *Vicia faba* (Linnaeus, 1753), an association of ɣ-tubulin with kinetochores and kinetochore fibres has been described after release from amiprophos-methyl treatments (antimicrotubular drugs) and on isolated chromosomes (Binarova et al. 1998), suggesting the possibility of MT nucleation at kinetochores.

It is also unclear why the metaphase I stage was not blocked. On the contrary, a tight chromosome mass with protuberant MT bundles was able to arrange chromosomes. Perhaps, when there are no bipolar-orientated chromosomes, there is no issue with ‘release of cohesion’ and the system of motor proteins and MT kinetochores can arrange chromosomes in 2R(2D)xR hybrids. At the beginning of anaphase I, univalents of 2R(2D)xR hybrids were arranged mainly into two groups and their kinetochore MTs cross-linked and focused. The single MT bundle was polymerised on kinetochores and inter-regional MT arrays were not present. Few interpolar microtubule bundles could be found in meiocytes, which were very thin compared to massively-wide MT bundles of kinetochores. Kinetochore MT bundles could be generated by the γ-TuRC-Augmin-mediated nucleation (Murata and Hasebe 2007, Lee et al. 2017). Chromosome separation and spindle poles focusing could occur through the functioning of kinesin–14A motor protein. The *Arabidopsis* kinesin–14A Atk1/AtKIN14a is involved in the assembly of the meiotic spindle and is needed for organising MTs at the two poles at metaphase and anaphase I and II (Chen et al. 2002) and the *divergent spindle–1(dv1)* gene encodes kinesin–14A that is specifically required for focusing the spindle pole to a fine point (Higgins et al. 2016).

A cell plate is formed after chromosome separation in a plant cell (De Storme and Geelen 2013). The cell plate is synthesised by a specialised structure called the phragmoplast, which consists of microtubules, actin filaments, membrane compartments and associated proteins (Murata et al. 2013, Smertenko et al. 2018). The phragmoplast forms between daughter nuclei during the transition from anaphase to telophase and originates from the remnants of the central spindle (Seguí-Simarro et al. 2007, Murata et al. 2013). The cases of *Arabidopsis* and *Physcomitrella paten* (Hedwig, 1801) moss show that MAP65 isotypes are localised in the mid-zone, where they stabilise the phragmoplast structure by cross-linking anti-parallel microtubules (Smertenko et al. 2000, Muller et al. 2004, Van Damme et al. 2004, Kosetsu et al. 2017), which presumes the need for anti-parallel microtubules to build phragmoplast.

MT bundles linking kinetochores were found at anaphase I of 2R(2D)xR between separated chromosome groups. Probably those MT arrays replaced inter-zonal MTs, as the bipolar spindle did not assemble in hybrids. Despite the absence of anti-parallel microtubules, a phragmoplast formed after chromosome separation. According to Smertenko et al. (2018), three zones form with distinct patterns of microtubule behaviour in the phragmoplast: the outer leading zone, the transition zone and an inner lagging zone. New MTs are formed in the outer leading zone; here cell plate assembly is initiated. A cell plate acquires the standard appearance in the transition zone through vesicle joining and migrating; the balance of microtubule polymerisation and depolymerisation is maintained here. In the inner lagging zone, the cell plate is practically formed and microtubules are depolymerised. As cytokinesis progresses and the phragmoplast array expands, microtubules are lost from the central region of the cell. Phragmoplast formation in hybrids, however, was delayed; it was found only in telophase and not in all meiocytes at the same time. Meiocytes were found where the leading edge of the phragmoplast reached the plasma membrane, but in a lagging zone, a cell plate was not formed. Nevertheless, phragmoplast was present in most cases, since 96% of meiocytes had tetrads at telophase II (Silkova et al. 2011). In wheat male meiosis, cytokinesis is successive, when each meiotic cell division is directly followed by a cytokinesis. Dyads are generated after meiosis I and tetrads are formed after meiosis II.

It is conceivable, perhaps, that replacing inter-zonal microtubules with the kinetochore ones in hybrids assumes another way/regulation of phragmoplast formation. Anti-parallel microtubules in normal meiosis constitute a phragmoplast “blank” and their absence in hybrids delays and interrupts phragmoplast expansion. However, meiocytes in meiosis II contained apparently normal bipolar spindles.

## Conclusions

Currently there is no universal model of spindle formation in plant meiosis. We discovered new structures in wheat and rye meiotic prophase and preprophase spindle. Based on it, we propose that chromatin– and pro-spindle-based cooperative mechanisms are needed to form a bipolar spindle in meiosis. Spindle assembly and pole marking in meiosis I take place similarly to mitosis. Probably, location sites of polar caps, for example, through ɣ-tubulin (Binarová et al. 2000, Canaday et al. 2004), retain memory to organise MTs in poles and their focusing.

Bipolar spindle in meiosis of asynaptic mutants of maize and rice haploids is formed regardless of the presence of bivalents (Chan and Cande 1998, Xue et al. 2019). On the contrary, in 2R(2D)xR hybrids, a bipolar spindle is not formed in the presence of bivalents. Therefore, cohesion release, but not the presence of bivalents, may affect bipolar spindle formation in meiosis. An anaphase promotion complex is activated only when a bipolar spindle is formed. Based on current data on the regulation of chromosome distribution during cell division, the sequence of possible events and their participants during chromosome segregation in the first meiosis of 2R(2D)xR amphihaploids can be represented as follows. At the prometaphase, the MAD2 protein in the complex with CDC20 is localised on kinetochores and MTs nucleate around the chromosome/kinetochore (nucleation depends on RanGTP gradient or aurora kinase). Only kinetochore MT bundles polymerise. In the absence of kinetochore tension due to its monopolar orientation, the MAD2/CDC20 complex is maintained. Aurora-kinase is localised on monopolar-orientated kinetochores. APC/C is not activated. Overall, the cohesion between the kinetochores and arms of sister chromatids does not cleave and cohesion is maintained. Thus, cohesion between univalent arms can be maintained due to the CDC20 co-factor related to CPC proteins, while cohesion in the centromeric region may be protected by SGO1. At anaphase I, univalents separate and their kinetochore microtubules are cross-linked and focused through the functioning of the kinesin–14A motor protein. At telophase I, kinetochore MT arrays replaced inter-zonal MTs and phragmoplast formation must be modified.
